# Impact of the Innate Inflammatory Response on ICU Admission and Death in Hospitalized Patients with COVID-19

**DOI:** 10.3390/biomedicines9111675

**Published:** 2021-11-12

**Authors:** Jorge Monserrat, Angel Asunsolo, Ana Gómez-Lahoz, Miguel A. Ortega, Jose Maria Gasalla, Óscar Gasulla, Jordi Fortuny-Profitós, Ferran A. Mazaira-Font, Miguel Teixidó Román, Alberto Arranz, José Sanz, Benjamin Muñoz, Juan Arévalo-Serrano, José Miguel Rodríguez, Carlos Martínez-A, Dimitri Balomenos, Melchor Álvarez-Mon

**Affiliations:** 1 Department of Medicine and Medical Specialities, Faculty of Medicine and Health Sciences, University of Alcalá, 28801 Alcalá de Henares, Spain; jorge.monserrat@uah.es (J.M.); alahoz1199@gmail.com (A.G.-L.); miguel.angel.ortega92@gmail.com (M.A.O.); 2 Ramón y Cajal Institute of Sanitary Research (IRYCIS), 28034 Madrid, Spain; angel.asunsolo@uah.es; 3 Department of Surgery, Medical and Social Sciences, Faculty of Medicine and Health Sciences, University of Alcalá, 28801 Alcala de Henares, Spain; 4 Department of Epidemiology and Biostatistics, Graduate School of Public Health and Health Policy, University of New York, New York, NY 10027, USA; 5 Cancer Registry and Pathology Department, Hospital Universitario Principe de Asturias, 28806 Alcalá de Henares, Spain; 6 Service of Internal Medicine and Immune System Diseases-Rheumatology, University Hospital Príncipe de Asturias, (CIBEREHD), 28806 Alcalá de Henares, Spain; jose.gasalla@uah.es (J.M.G.); jarranzhupa@gmail.com (A.A.); jsanz.hupa@salud.madrid.org (J.S.); benmunozc@hotmail.com (B.M.); jarevalo454@gmail.com (J.A.-S.); jmiguel.rodriguez@uah.es (J.M.R.); 7 Hospital Universitari de Bellvitge—Universitat de Barcelona, 08907 L’Hospitalet de Llobregat, Spain; ogasulla@bellvitgehospital.cat; 8 Campus Nord, Universitat Politècnica de Catalunya, 08034 Barcelona, Spain; jordi.fortuny.profitos@estudiant.upc.edu (J.F.-P.); miguel.teixido.roman@estudiant.upc.edu (M.T.R.); 9 Departament d’Econometria, Estadística I Economia Aplicada—Universitat de Barcelona, 08007 Barcelona, Spain; ferranmazaira@gmail.com; 10 Department of Immunology and Oncology, Centro Nacional de Biotecnología/CSIC, 28006 Madrid, Spain; cmartineza@cnb.csic.es (C.M.-A.); dbalomenos@cnb.csic.es (D.B.)

**Keywords:** SARS-CoV-2, ICU, innate inflammatory response, COVID-19, cytokines

## Abstract

Objective: To describe the capacity of a broad spectrum of cytokines and growth factors to predict ICU admission and/or death in patients with severe COVID-19. Design: An observational, analytical, retrospective cohort study with longitudinal follow-up. Setting: Hospital Universitario Príncipe de Asturias (HUPA). Participants: 287 patients diagnosed with COVID-19 admitted to our hospital from 24 March to 8 May 2020, followed until 31 August 2020. Main outcome measures: Profiles of immune response (IR) mediators were determined using the Luminex Multiplex technique in hospitalized patients within six days of admission by examining serum levels of 62 soluble molecules classified into the three groups: adaptive IR-related cytokines (*n* = 19), innate inflammatory IR-related cytokines (*n* = 27), and growth factors (*n* = 16). Results: A statistically robust link with ICU admission and/or death was detected for increased serum levels of interleukin (IL)-6, IL-15, soluble (s) RAGE, IP10, MCP3, sIL1RII, IL-8, GCSF and MCSF and IL-10. The greatest prognostic value was observed for the marker combination IL-10, IL-6 and GCSF. Conclusions: When severe COVID-19 progresses to ICU admission and/or death there is a marked increase in serum levels of several cytokines and chemokines, mainly related to the patient’s inflammatory IR. Serum levels of IL-10, IL-6 and GCSF were most prognostic of the outcome measure.

## 1. Introduction

SARS-CoV-2 is a complex pathogen with a high rate of infectivity and transmissibility [1,2]. Respiratory droplets are the most common form of dissemination, although aerosols and the fecal–oral route are also possible modes of transmission [3]. Via recognition of the angiotensin-converting enzyme 2 receptor (ACE-2) by the viral spike (S) protein, SARS-CoV-2 enters the cell, where it replicates its own genome and assembles new viruses which spread throughout the body [4]. Interestingly, ACE-2 is widely expressed in a wide variety of tissues, and therefore SARS-CoV-2 is able to infect almost any type of cell [5]. Nonetheless, the lungs are the hardest-hit organ, particularly type 2 alveolar epithelial cells [6]. Immune cells are central players in COVID-19.

An adequate innate and adaptive immune response (IR) is essential to limit the spread of the virus and the damage it causes [7]. Innate immunity is an individual’s first line of defense, and its main components are myelomonocyte cells, natural killer (NK) cells, neutrophils and eosinophils. These cells recognize SARS-CoV-2 pathogen-associated molecular patterns (PAMPs) through their Toll-like receptors (TLR), inducing an adequate effector response involving the secretion of different soluble mediators including interferons (IFN), interleukins, chemokines and growth factors [8]. Adaptive immunity is mediated a few days post infection by T lymphocytes, including helper (Th), T regulatory (Treg), T cytotoxic (Tc) subsets and B cells, orchestrating a specific targeted effector response against SARS-CoV-2 [9]. This lymphocyte activation is also followed by the secretion of cytokines, chemokines and growth factors. The immune system response to SARS-CoV-2 infection is a complex balance between an efficient effector response and the induction of a bystander uncontrolled local systemic inflammation causing tissue damage [10]. In severe cases of COVID-19, an abnormal IR may lead to a hyperinflammatory state whereby intense cytokine production is triggered, known as a “cytokine storm”, with severe clinical implications for the patient [11,12,13]. This impaired immune system control and dysregulated cytokine production is crucial to fully understanding the pathogenesis of severe COVID-19 [14].

In this context, several studies have shown the value of cytokine profiles as predictors of COVID-19 severity. So far, modified levels of classic pro-inflammatory cytokines such as interleukin (IL)-6 and tumor necrosis factor-α (TNF-α), as well as of typical anti-inflammatory cytokines such as tumor growth factor β (TGF- β) or IL-10, have been associated with an increased disease severity and risk of mortality [15,16,17]. Furthermore, abnormally increased levels of other soluble inflammatory molecules such as chemokines and growth factors, and of endothelial- and platelet secreted molecules and neuron specific enolase, have been also described in patients with severe COVID-19 [18]. Nevertheless, we still lack a comprehensive analysis of a wide spectrum of IR mediators. The complex regulation of production, multicellular origin and interplay between cytokines and other signaling molecules supports the simultaneous study of these molecules to establish which ones play the most important role in COVID-19 progression. In parallel, an imbalance among immune cell populations has been shown to contribute to the pathogenesis of COVID-19 [16]. Accordingly, we have described that a reduced lymphocyte/leukocyte ratio together with an elevated total leukocyte count is an important marker of COVID-19-related mortality [19]. Other authors have also highlighted the clinical relevance of lymphopenia leading to defective antiviral and regulatory immunity, accompanied by sustained elevation of cytokine-producer cells [20,21].

The aim of our study was to examine the role of a broad spectrum of immune-inflammatory soluble molecules, including cytokines, chemokines, growth factors and vascular inflammatory mediators, as predictive and prognostic markers of COVID-19 outcome to assess their potential implications for case fatality in hospitalized patients.

## 2. Patients and Methods

### 2.1. Study Population and Definitions

This study was designed as an observational, analytical, retrospective cohort study with longitudinal follow-up. The study population consisted of 287 patients diagnosed with COVID-19 according to the criteria established by the WHO, based on the results of a real-time reverse transcription polymerase chain reaction (RT-qPCR) test on a nasopharyngeal sample. For each participant, consecutive blood samples were submitted to the Biochemistry Service of the Hospital Universitario Principe de Asturias from 24 March to 8 May 2020 and patients were followed until 31 August 2020 [22]. Inclusion criteria for patients admitted to the Hospital Universitario Principe de Asturias (HUPA) were (1) respiratory rate ≥ 30 breaths/min, (2) SpO2 ≤ 94% while breathing ambient air, and (3) opacities detected in a chest X-ray as defined by the Diagnosis and Treatment Protocol for Novel Coronavirus Pneumonia (6th interim edition) [23]. We obtained demographic data and information concerning comorbidities from electronic health records.

Standard treatment (ST) and clinical management was carried out according to established protocols (Supplementary Material File SI). On admission, patients received oxygen support through a low-flow nasal cannula to maintain SpO2 > 90%. Patients with increased oxygen needs were switched to a high-flow oxygen mask (Venturi mask up to 50% FiO2). Mechanical ventilation (MV) was provided only to patients admitted to the ICU. In addition, blood samples were collected within the first six days of hospital admission. The target of our analysis was defined as ICU admission and/or Exitus (ICU/Exitus). A total of 337 blood samples were collected, of which 103 were obtained on the first day, 55 the second, 52 the third, 52 the fourth, 53 the fifth, and 22 the sixth. In 247 patients, only one blood sample was collected. In the 40 patients in whom two or more samples were obtained, only the first sample was considered. Serum from 14 age-matched healthy individuals was used to set up controls (denoted HC).

### 2.2. Determination of Immune Response Mediators

We determined serum levels of IL22, soluble (s) sCD40 ligand (L), IL17EIL25, sCD30, sgp130, IFNa2, IL13, IL9, TNFb, IL5, IL4, IL17A, sIL4R, IL17F, IL10, sIL1RI, IFNg, TGFa, sIL2Ra, IL3, PDGFABBB, PDGFAA, sVEGFR1, FGF2, FLT3L, sVEGFR2, sVEGFR3, GMCSF, VEGFA, IL2, EGF, GCSF, IL7, MCSF, MDC, IL12p70, IL18, FRACTALKINE, IL27, IL12p40, MIP1a, MIP1bota, IL1b sIL6R, TNFa, sRAGE IL1RII, IP10, IL1a, MCP1, IL6, IL1RA, RANTES, IL15, sTNFRI, sTNFRII, MIG, and IL8. These soluble molecules were classified into three groups determined by their main biological activity as innate inflammatory and adaptive IR cytokines and growth factors, as shown in Supplementary Material File SII. For this purpose, aliquots of serum were obtained from peripheral blood in a dry tube by centrifugation at 2000 rpm for 20 min and kept at −80 °C until analysis.

Multiplex assays were performed using the Luminex^®^ technique with a high sensitivity kit (Milliplex MAP kit) from Merck laboratory (Darmstadt, Germany). For cytokines, microspheres were used, each one excited by red (635 nm) and green (525 nm) LEDs and classified according to the different amounts of fluorescence emission depending on the analyte to be studied. Plates (96-well) were incubated for 16–18 h with the antigen for binding to the capture antibody of each microsphere. After incubation, the biotinylated detection antibody for each cytokine was added. Finally, a streptavidin–phycoerythrin complex (Strep-PE) was added to bind the detection antibody. The plate was read on a MAGpix system (Merck).

Using the standard curve, the Merck analysis program (Analyst) calculated the concentration of each cytokine of interest using the mean fluorescence intensity (MFI). For each cytokine analyzed according to the protocol, detection limits were established.

### 2.3. Statistical Analysis

To assess the effect of the secretion of signaling molecules on the risk of a fatal outcome (ICU/Exitus) of COVID-19 at the time of hospital admission, we designed a four-step empirical strategy.

First, we performed a one-on-one analysis to assess the impacts of each standalone marker. Percentage expression and the level of each mediator were compared with HC. In a COVID-19 patient, an elevated level of a given molecule was defined as at least twice the average level for the group. To predict ICU/Exitus, we calculated the odds ratio for each molecule among patients in whom the molecule was elevated versus in the rest of the patients.

Secondly, we tested the extent to which factors such as gender, age, medical records, comorbidities, oxygen saturation, and other soluble molecules emerged as significant in a univariate analysis for their inclusion in a multivariate analysis. To this end, we built 4495 logistic regression models, using as explanatory variables a risk score model based on electronic health records [16] and all the possible combinations of three mediators out of the selected molecules. Each selected molecule was thus used as an explanatory variable in 465 models, and combined with the risk score and two other molecules, which were different in every model. We constructed Bayesian logistic models, whereby no fixed distribution was assumed on the parameters, using the brms package available in R and with no priors to avoid introducing any bias. For each model, we assessed individual contributions in terms of AUCs of the cytokines, and ranked the most relevant based on their average AUC contribution. To avoid multi-collinearity problems, we analyzed the crossed correlation of the molecule and estimated the variation inflation factor (VIF).

In the third step, we built a final model with the molecules that had more predictive and were statistically robust for the inclusion of other molecules and medical record data, and estimated the relative importance of these molecules in the final model using SHAP (SHapley Additive ExPlanation) values [24,25]. Three models were prepared using Bayesian GLM estimates. In the first model, the best contributor of each group—that is, for each type of molecule, the one showing a higher individual AUC contribution based on the multivariate analysis—was selected according to biological functional activity. In the second model, for each patient we selected the molecules in each group that showed the largest deviation versus levels in healthy volunteers. Hence, the model tested whether, within a group of molecules, the most predictive factor was that showing the maximum relative secretion out of any of the mediators belonging to that group. The third model estimated the average deviation from HC levels of each group. This model tested whether the most important predictor was not a given cytokine or growth factor (as in the best model contributor), or the largest deviation with respect to healthy volunteers (as in the largest deviation model), but the average response of the patient.

Finally, as a robustness check, we repeated all analyses using only data derived from blood samples taken within the first three days of admission. This way, we could confirm that a time span of six days, which allowed for a larger sample size, did not compromise the results. The statistical treatment of data through univariate and multivariate analysis is described in Supplementary Material File SIII.

### 2.4. Ethics and Approval

This study was conducted according to basic principles of ethics (autonomy, harm avoidance, benefit, and distributive justice). The protocol was in line with the standards of Good Clinical Practice and the principles of the last Declaration of Helsinki (2013) and the Oviedo Convention (1997). Ethics committee approval was obtained from the University Hospital Príncipe de Asturias (HUPA-04062020).

## 3. Results

### 3.1. Clinical Characteristics of the Patient Population

Table 1 shows the demographic and clinical characteristics of the 287 COVID-19 patients included in this study. Of these, 62 patients (22.2% of the total group) were admitted to the ICU, and 37 (12.9%) died. There were no significant differences in the age and sex of patients with severe COVID-19 who progressed to ICU/Exitus and those who did not. In the ICU/Exitus group, there were significantly higher percentages of patients with the comorbidities cor pulmonale, hypothyroidism, obesity, acute and chronic renal failure and dementia. Significant differences were also detected in Charlson and Elixhauser indices between both groups of patients (Charlson: 0.02228 and Elixhauser: 0.01412). Blood oxygen saturation levels were also significantly worse in the ICU/Exitus group.

### 3.2. Severe COVID-19 Causes Dysregulation of Innate Inflammatory and Adaptive IR Cytokines and Growth Factors

First, we measured serum cytokine and growth factor levels in the severe COVID-19 patients and HC (Supplementary Material File SIV). Out of the 19 adaptive IR-related cytokines quantified, 15 were detected at elevated levels in more than 90% of the patients. Elevated serum levels of IL22, sCD30, IL10, IL17 F and sIL1R1 were found in 47%, 61.7%, 89.9% and 14.3%, respectively. High levels of 25 innate inflammatory IR-related cytokines were also detected in more than 90% of the severe COVID-19 patients, but those of IL12p70 and sRAGE were found in 78% and 88.5% of the patients, respectively. Eleven of the sixteen growth factors measured were detected at elevated levels in more than 90% of patients. IL3, sVEGFR1, GMCSF, IL2 and GCSF were detected at elevated levels in 20.2%, 39%, 18.5%, 86.4% and 85%, respectively.

**Figure 1:**
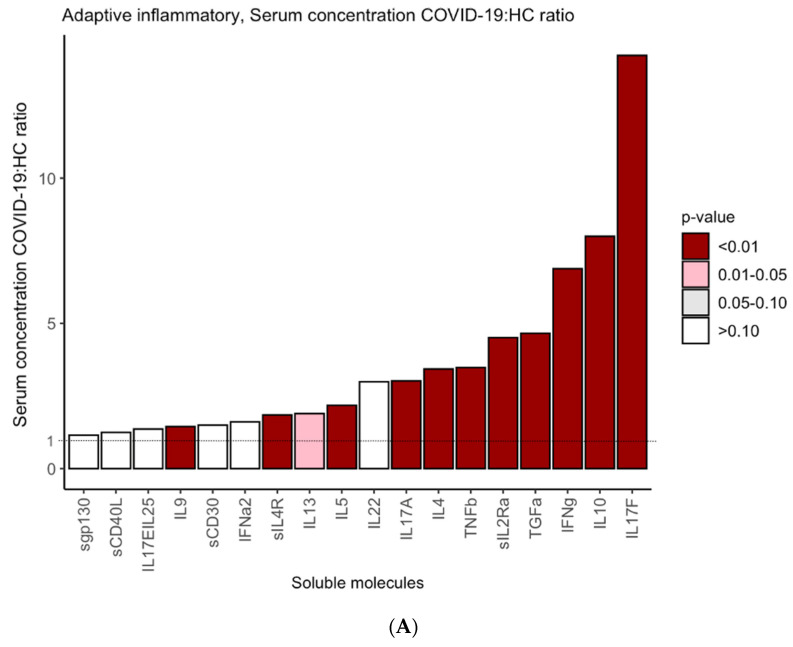

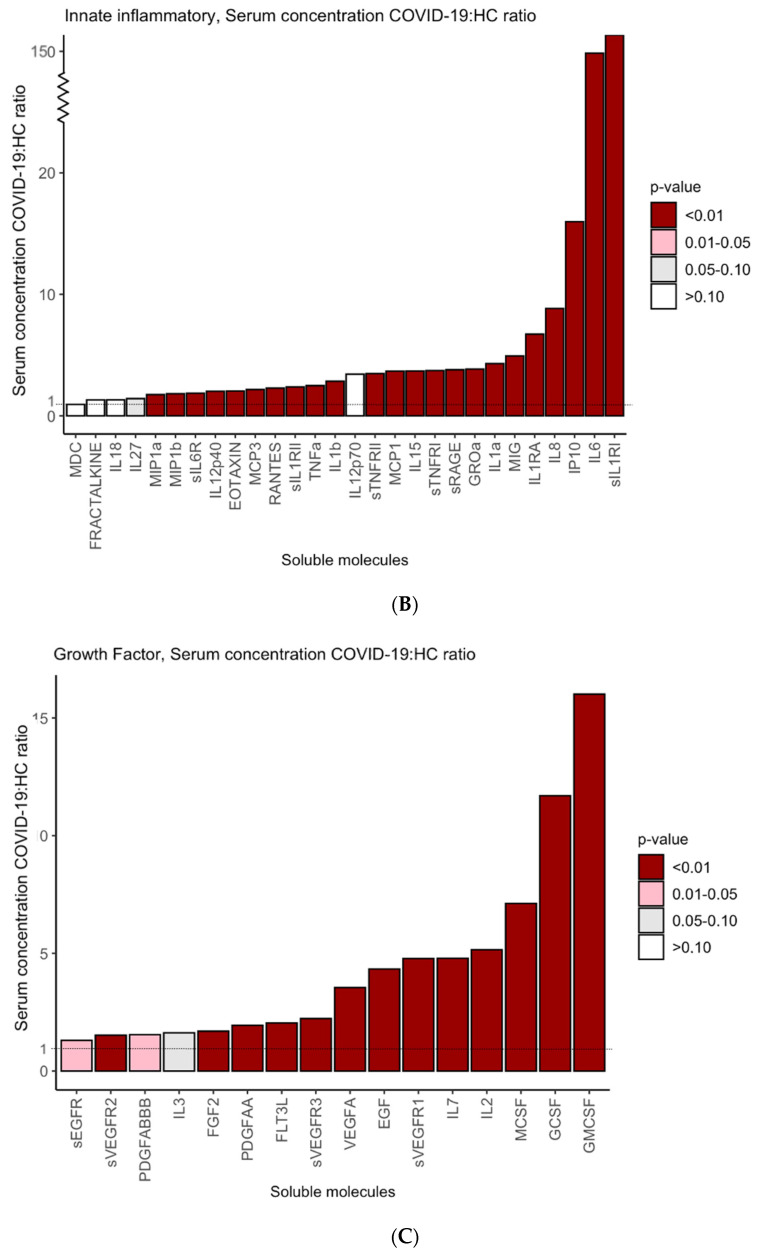
Serum levels of cytokines and growth factors in COVID-19 patients and HC. Y axes indicate the ratios of concentrations of each cytokine in the COVID-19 patients to those in HC. Ratios for adaptive IR-related cytokines (**A**), innate inflammatory-related IR cytokines (**B**) and growth factors (**C**). The vertical bars represent the mean of the COVID-19:HC ratios for the indicated molecule. The intensity of color denotes significant differences between serum levels of each cytokine in the COVID-19 patients and HC.

Next, we compared serum levels of the markers in the COVID-19 patients and HC. As shown in Figure 1A, significantly higher levels of the adaptive IR cytokines IL17F, IL10, IFNg, TGFa, sIL2Ra, TNFb, IL4, IL17A, IL5, sILR, IL9 (all *p* < 0.01) and IL13 (*p* < 0.05) were detected in the patients than in healthy controls. Similarly, levels of innate IR cytokines (IL6, IP10, IL8, IL1RA, MIG, IL1a, GROa, sRAGE, STNFRI, IL15, MCP1, sTNFRII, IL1b, TNFa, sIL1RI, sIL1RII, RANTES, MCP3, EOTAXIN, IL12p40, sIL6R, MIC1b and MIC1a) were significantly higher (*p* < 0.01) in the patients than in controls (Figure 1B) and those of the molecules GMCSF, GCSF, MSCF, IL2, IL7, sVEGFR1, EGF, VEGFA, sVEGFR3, FLT3L, PDGFAA, FGF2, sVEGFR2 (all *p* < 0.01) and PDGFABBB and sEGFR (both *p* < 0.05) were also significantly higher in the COVID-19 patients.

### 3.3. Serum Cytokine/Growth Factor Levels Are Robust Predictors of ICU Admission and/or Death in Hospitalized COVID-19 Patients

**Figure 2:**
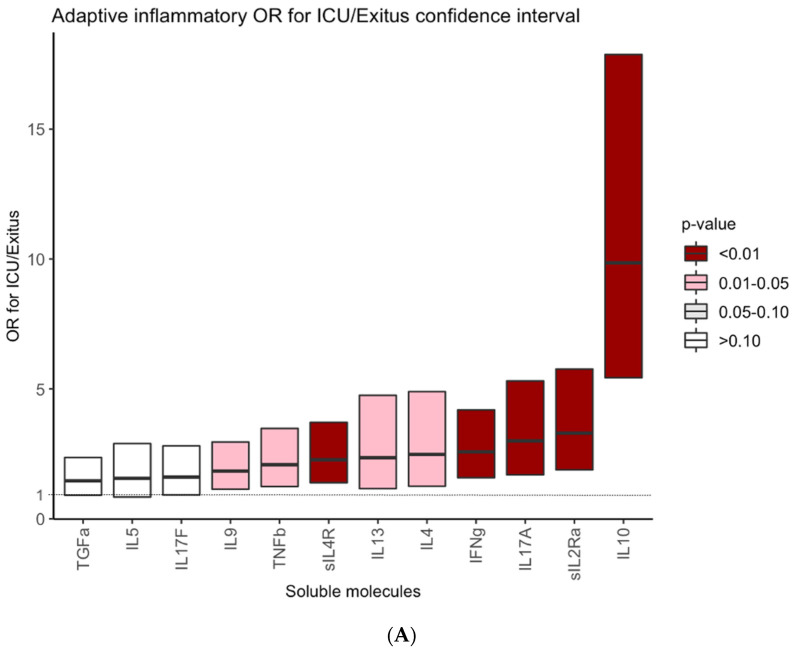

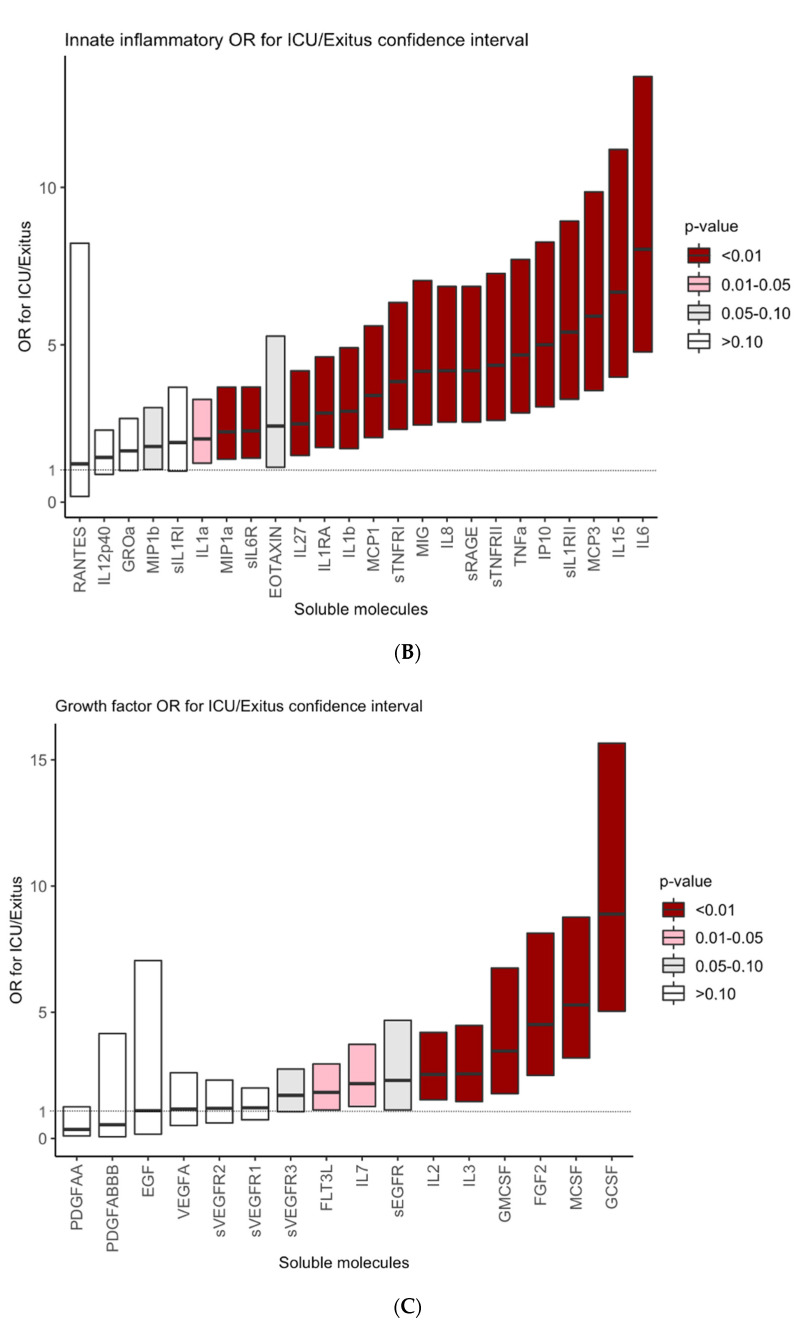
Odds ratios for ICU/Exitus of serum levels of adaptive IR-related cytokines (**A**), innate inflammatory IR-related cytokines (**B**) and growth factors (**C**) in COVID-19 patients. Y axes show the odds ratio of increased concentrations of each marker for ICU/Exitus. The intensity of the color grades the level of the significance of the odds ratio.

We then went on to explore the predictive value of serum marker levels for the ICU/Exitus outcome of the COVID-19 patients. We centered our analysis on the 52 molecules whose serum levels were found to vary significantly between the COVID-19 patients and HC. As shown in Figure 2, the odds ratio for ICU/exitus of 39 out of the 52 molecules was significant and the odds ratio of 35 of them was higher than two. Interestingly, IL10, GCSF, IL6, IL15, MCP3, sIL1RII, MCSF and IL10 returned the higher odds ratios (9.9, 8.9, 8, 6.7, 5.9, 5.4, 5.3 and 5, respectively).

It is important to highlight that these results are robust at restricting the target outcome only to “Exitus”. Specifically, the significance of only six (IL17F, TGFa, SEGFR, sVEGFR3, IL4 and MIP1B) out of the 52 molecules changed when considering “Exitus” as the target (four were significant for the target outcome ICU/Exitus and not for Exitus, and two were significant for Exitus and not ICU/Exitus) (Supplementary Material File SV).

### 3.4. A Subset of Mediators Predicts the Progression of Severe COVID-19 to ICU Admission and/or Death

According to our univariate analysis, the concentrations of 39 molecules varied between patients progressing to ICU/Exitus and the rest of the patients.

Molecules could then be ranked according to their capacity and significance of this capacity to predict progression to ICU/Exitus of COVID-19 patients. To avoid multi-collinearity problems, we analyzed crossed correlation of serum concentrations of the 39 markers. It emerged that the behavior of 11 markers showed at least 85% correlation with that of another one. These molecules were classified based on these correlations among pairs into three groups (>95%, <95%, >85%, respectively): TNFb, IL13, IL4 and TNFa; IL1b, FGF2, IL1a, IL17A, and IL15; and STNFRI and STNFRII. We then examined the predictive value for ICU/Exitus of serum levels of these molecules in addition to patient age, gender, comorbidities and oxygen saturation [24] (Supplementary Material File SVI). For each of these groups, only the molecules with the higher individual AUCs were chosen, namely, TNFa, IL15 and STNFRII. This provided a final set of 31 molecules.

Next, 4495 different Bayesian GLM models of all possible combinations of 3 molecules of the 31 selected were constructed. We estimated AUC gain of the prognostic value of each set of molecules over that calculated with the clinical score described previously. Therefore, each molecule was used in 465 models (Table 2). Furthermore, for each molecule, we calculated the percentage of combinations that resulted in a significantly increased prognostic value of the clinical score and average AUC gained.

Our criterion for the final selection of variables was all those molecules that featured a significance of more than 95% in the models as well as an average AUC gain over two points. As can be seen in Table 2, 10 molecules fulfilled these criteria. Those 10 molecules were considered the most robust across all models and were selected for the final model.

It is important to note that for this selection, multi-collinearity between serum cytokine levels was not a problem. The average VIF was 1.1 and the maximum was 2.3.

### 3.5. Innate-Inflammatory IR-Related Cytokines and Growth Factors Play an Important Prognostic Role in Patients with Severe COVID-19

**Figure 3:**
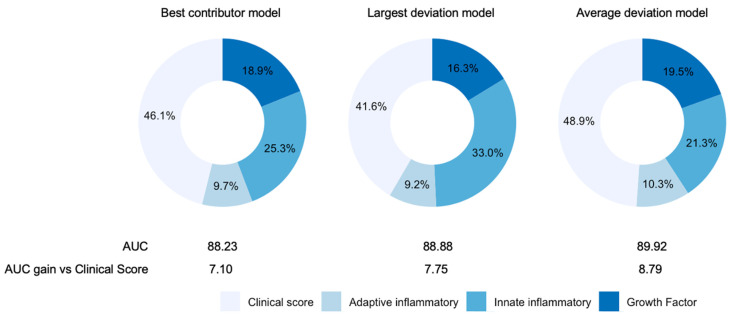
Contributions of the selected molecules to the final models of prediction of ICU admission and/or death in COVID-19 patients. In addition to the different combinations of molecules included in the three models, the AUC of the prognostic value of clinical score (age, sex, comorbidities and blood oxygen saturation) was calculated. Percentages represent the relative importance of the four different components of variables in each model estimated using SHAP values.

Finally, after checking the robustness of 10 molecules adjusted for other effects, a final model was built to assess the relative contribution of each molecule and their combinations to the prognosis of severe COVID-19. Three models based on GLM estimates are presented here (Figure 3). The first model provided the best contributor of the three groups of molecules (innate inflammatory IR cytokines, adaptive IR cytokines and growth factors); that is, the molecules showing a higher individual AUC contribution based on the multivariate analysis (Table 2). The figure shows that the best contributors were IL10, GCSF and IL6. In this model, including serum levels of these three molecules increased the AUC by 7.1 to give a prognostic value of 88.23 for ICU/Exitus versus the clinical score. Remarkably, according to SHAP values, these three molecules accounted for more than 50% of the model. We should also highlight that if the best contributors were not restricted to the top molecule in each group, such that the top three molecules combined yielding the highest AUC were considered, IL10 would be replaced with sIL1RII and the AUC would change from 88.2 to 89.8.

The second model consisted of identifying for each patient the signaling molecule in each group that showed the greatest deviation versus HC. Hence, the model tested whether, within a group of molecules, the most predictive factor was the maximum relative serum level of any of those belonging to the group. In this model, we also observed that the individualized selection of three molecules according to the indicated criterion increased the AUC by 7.75 to attain a value of 88.88 as the prognostic value for ICU/Exitus. In each patient, the relative contribution of the best innate inflammatory IR cytokines (IL6, IL15, sRAGE, IP10, MCP3, sIL1RII and IL8) to the model was 33%.

The third model estimated the average deviation from HC of serum levels of each group of molecules. Hence, it did not test whether the most important predictor was a given molecule (as did the best contributor model) or those that showed the largest deviations; rather, it tested the average response. Average deviation was weighted by the individual AUC contribution, as may be found in Table 2. By including the median of the variation shown by the molecules in each group, the AUC was enhanced by 8.79. The relative prognostic capacity of the innate inflammatory IR cytokines was also the highest in this model.

The robustness of the predictive power of quantification of the selected molecules was also observed using the oxygen saturation parameters of the patients obtained in the 24 h period that included the time of blood withdrawal (Supplementary Material File SVI). Figure 3 illustrates the relative importance estimated using SHAP values of each group of molecules in the three models. As a final robustness check, we determined the extent to which our results would be robust to a modification in the time of blood sample collection, by constraining this interval to only the first three days of admission. This reduced the sample size from 287 patients to 195. Nine signaling molecules were found to show significant predictive power. These were the same as for the unrestricted sample, with the exception that IL8 and SILR1II were replaced by IL3. The AUCs and relative contributions of the different groups in the models were in line with our previous results (see Supplementary Material File SVII).

## 4. Discussion

More than a year after the SARS-CoV-2 pandemic was declared, the best individual treatment for SARS-CoV-2 remains elusive and while the number of vaccinated people is growing daily, it is difficult to predict when the pandemic will end [26]. The medical management and treatment of severe COVID-19 demands knowledge of its prognosis in an individual patient. Further, the anxiety and discomfort generated by the unpredictable course of severe COVID-19 mandates that potential biomarkers of the disease need to be established.

The immune system plays a critical role in the pathogenesis of SARS-CoV-2 infection. Hence, targeting the immune system and the dysregulation of its major components is a good strategy to discover potential therapies for severe cases of COVID-19 [27]. Several studies have shown the clinical relevance of patient profiling to predict the outcome of COVID-19 [28,29,30] and to distinguish SARS-CoV-2 infection from other respiratory conditions [31]. In this work, we explored the predictive value for the risk of fatality in patients with severe COVID-19 of a comprehensive number of immune-inflammatory soluble signaling molecules measured within the first six days of hospitalization. We found that increased serum levels of IL10, a key regulatory cytokine of the immune response, the growth factors GCSF and MCSF, and the innate inflammatory IR mediators IL-6, IL-15, sRAGE, IP10, and MCP3 showed strong prognostic capacity for the progression of severe COVID-19 to ICU admission and/or death. Our results are consistent with the findings of another study in which important alterations in innate and adaptive IR cytokines, as well as growth factors, were associated with COVID-19 severity [32].

Our study highlights the link between increased levels of signaling molecules of the innate inflammatory system and a poor clinical course of COVID-19. Among these molecules, IL-6 and IL-15 were the major contributors to the fatality risk in patients with severe COVID-19. The cytokines IL-6 and IL-15 are secreted by different cells of the immune system, mainly by those involved in the innate immune response but also by epithelial and mesenchymal cells. IL6 is a critical proinflammatory cytokine. IL6 and IL15 have regulatory effects on innate and adaptive responses and also mediate hematopoiesis [33,34]. Interestingly, elevated IL15 levels have been associated with dysfunction of IR phagocytic cells and an ineffective response to viral infections. IL15 has been also associated with the induction of IR. Reports exist of IL-6 and IL-15 upregulation in patients with severe presentations of COVID-19 [35]. Moreover, other authors have reported a direct correlation between IL-6 levels and inflammasome hyperactivation, typically occurring in severe cases of COVID-19 [36]. We were able to relate higher levels of IL-8 to COVID-19 fatality, but with limited prognostic value, therefore not supporting the proposed role of IL-8 as a potential biomarker of prognosis in COVID-19 patients [15,37,38]. The link between elevated levels of molecules of the innate inflammatory response and a worse course of severe COVID-19 was also supported by the prognostic value of the chemokines IP10, MCP3 and sRAGE, and the cytokine receptor sIL1RII. These results expand the observation that sRAGE may be involved in worsening of asymptomatic COVID-19 and IP10 and MCP3 in symptomatic expression of the disease [39,40]. In the case of sIL1RII, there were no reported alterations, although blocking the binding of this receptor to its ligand IL-1 seems a potential target to prevent COVID-19′s complications [41]. The prognostic value of IL-6, IL-15, sRAGE, IP10, and MCP3 to predict a poor COVID-19 outcome is consistent with the observation of an exacerbated innate inflammatory response in patients with severe disease [42]. The relevance of the innate inflammatory response is effectively supported by the confirmation that IL6 and IL10 are the two cytokines with the highest capacity to predict a fatal course of severe COVID-19.

We detected elevated levels of IL-10 in our group of patients with severe COVID-19. IL-10 is a key regulatory cytokine of innate inflammatory and adaptive immune responses produced by different lymphocyte populations. Tregs are a critical source of this cytokine [43]. IL10 has suppressor regulatory effects upon effector immune system cells but may also adversely regulate pro-inflammatory cytokines [44,45]. IL-10 may play a role in COVID-19 disease progression because of its behavior as an immune activating/proinflammatory agent. This cytokine stimulates the production of other mediators of the cytokine storm and simultaneously targets cellular exhaustion, possibly affecting virus tolerance, leaving cells in a refractory state with no response to virus stimulation. Our results are consistent with those of studies that have compared groups of patients with mild and severe COVID-19. In such studies, positive correlation has been observed between IL-6 and IL-10 levels and disease progression and severity [46]. Some investigations have detected much higher IL-6 and IL-10 levels in patients with critical COVID-19 than in those with severe COVID-19 [17]. In critical COVID-19 patients, a rise in serum IL10 levels occurs subsequently to elevated levels of innate inflammatory molecules IL-1β, IL-6, IL-8, and sTNFR1 during the course of the infection [47]. It should be noted that studies have specifically mentioned the role that IL-8 has in normal and pathological conditions in human bronchial epithelial cells, and therefore its possible potential role in infectious pathologies such as COVID-19 [48,49,50]. Our data revealed increased IL10 levels in severe COVID-19 and even higher levels in patients showing an unfavorable disease course. Furthermore, data comparing stable and ICU patients indicated lower IL-10 concentrations in the former, revealing that while other cytokines involved in innate immunity, such as IL-1β, IL-6, IL-8, and sTNFR1, show high secretion levels in all COVID-19 patients, IL-10 levels are significantly higher in critical patients. It has also been reported that IL-10 and other molecules combined (IL-1RA and CCL5) are especially helpful as prognostic biomarkers [51]. Although the main role of IL-10 is its anti-inflammatory role in the lung, its higher production by Tregs has been correlated with a harsher COVID-19 phenotype [52] and poorer outcomes, making this cytokine an interesting target for more accurate immunotherapy [53]. There is also evidence that IL-10 elevation is a consequence of a severe proinflammatory setting [54]. As a result, some authors advocate the IL-6:IL-10 ratio for a rapid diagnosis and to drive clinical management [55]. For example, this ratio may be used as a basis for the decision to withdraw mechanical ventilation and initiate a different regimen of clinical care [56]. Liu et al. [57] observed that both IL-6 and IL-10 levels were significantly increased in patients with critical COVID-19 compared to those with moderate or severe disease. Nevertheless, it is not clear if overactivation or suppression of IL-10 could improve clinical severe manifestations of COVID-19; more knowledge related to this topic is needed [58]. Additionally, each patient must be treated individually for their particular symptoms. This issue is particularly relevant now that it is known that pleiotropic IL-10 is subjected to multiple polymorphisms that may determine a predisposition for several chronic diseases such as asthma, rheumatoid arthritis or inflammatory bowel disease [59].

Increased serum levels of GCSF and MCSF were here related to the risk of ICU admission or death in our severe COVID-19 patients. GCSF is a key regulator of neutrophil production and activity, also influencing T cell function and dendritic cell activation [60]. Neutrophils are important players in the IR, performing phagocytic functions against SARS-CoV-2. However, neutrophils are also responsible for the production of neutrophil extracellular traps (NETs), which have been associated with coagulation disorders occurring in patients with severe COVID-19 [61]. The increase in GCSF produced may be related to an enhanced inflammatory response and particularly to neutrophil production and function, which may be associated with the pathophysiological complications of SARS-CoV-2 infection. In contrast, MCSF is a central cytokine of the macrophage-mediated immune response [62]. As with neutrophils, macrophage hyperactivation is essential to explain the pathological inflammation that occurs in COVID-19 [63]. Thus, studies have shown significant alterations in both GCSF and MCSF related to COVID-19 severity [47], supporting the important role of these two factors in the immune response against SARS-CoV-2.

The average age of the present patients was 63.9 years and 65% were male. Some age-related events such as inflamm-aging may play a central role in the appearance of cytokine storms [64]. In addition, studies have shown a greater severity of COVID-19 in men than women [65] and both sexes have also been reported to show a different profile of inflammatory system components [66,67]. As future work, we propose analyzing cytokines that are only affected in women with severe COVID-19 to establish a proper inflammatory profile in this patient subset and determine the role of MMPs.

Our findings indicated that patients with severe COVID-19 who progress to ICU admission and/or death are characterized by a markedly disturbed IR, mainly involving elevated serum levels of IL6, IL15, sIL1RII, sRAGE, IP10 and MCP3 in the first week of hospitalization. This overstimulated IR was already observed within the first three days of admission. Furthermore, the immune dysregulation observed in severe COVID-19 patients with a poor prognosis was also associated with increased GCSF and MCSF levels, which are essential growth factors for IR cells. In contrast, increased serum levels of cytokines of the adaptive IR do not seem to be of prognostic value for severe COVID-19 patients. An exception is IL10, which can be considered a bridge between the innate and adaptive IR. Accordingly, the innate IR seems to play a critical role in the pathogenesis of severe COVID-19 progression to ICU admission and/or death.

## 5. Conclusions

The results of our study indicated that serum levels of the molecules IL6, sIL1RII and GCSF are of high prognostic value for severe COVID-19 in the first week of hospitalization. Our model defined and predicted the immune response of a given patient to COVID-19. This type of model pursues a personalized and effective management approach to COVID-19 in this time of the pandemic.

## Figures and Tables

**Table 1:** Description of the COVID-19 patients included in the study. Demographic and clinical characteristics (mean and standard deviation) of the patients who survived and were not admitted to the ICU and those who were admitted to the ICU and/or died are shown.

	No ICU/Exitus (*n* = 225)	ICU/Exitus (*n* = 62)	*p*-Value
Age (years), mean (SD)	63.8 (12.2)	64.3 (13.7)	0.816
Gender, female/male	35%/65%	35%/65%	1
Oxygen saturation on hospital admission			<0.001
Extremely low (<80%)	1%	32%	
Low (80–89%)	12%	27%	
Medium (90–94%)	62%	37%	
Normal (>94%)	25%	3%	
Charlson Index	0.8 (1.1)	1.3 (1.4)	0.022
Elixhauser Index	2 (1.9)	2.8 (2.1)	0.014
Hypertension	45.3%	38.7%	0.176
Metabolic-endocrine diseases	30.2%	37.1%	0.152
Diabetes	21.8%	29.0%	0.116
Hypothyroidism	6.7%	0.0%	0.018
Obesity	8.9%	16.1%	0.049
Heart diseases	13.3%	35.5%	<0.001
Cor pulmonale	6.7%	24.2%	<0.001
Ischemic heart disease	5.8%	6.5%	0.421
Congestive heart failure	4.4%	8.1%	0.128
Respiratory diseases	14.7%	14.5%	0.488
Asthma	8.0%	6.5%	0.342
COPD (chronic obstructive pulmonary disease)	6.2%	8.1%	0.303
Other	2.2%	1.6%	0.383
Kidney diseases	9.3%	30.6%	<0.001
Acute kidney failure	6.2%	27.4%	<0.001
Chronic kidney disease	4.9%	9.7%	0.079
Autoimmune and rheumatic diseases	8.4%	9.7%	0.380
Rheumatoid arthritis	1.3%	3.2%	0.157
Spondyloarthritis	1.3%	1.6%	0.434
Psoriasis	1.3%	0.0%	0.180
Vasculitis	4.4%	6.5%	0.258
Hematologic malignancies	2.2%	1.6%	0.383
Leukemia	0.4%	1.6%	0.165
Lymphoma	0.9%	0.0%	0.228
Multiple myeloma	0.9%	0.0%	0.228
Solid tumors	1.3%	1.6%	0.434
Bowel cancer	0.4%	1.6%	0.164
Breast cancer	0.0%	0.0%	0.500
Lung cancer	0.9%	0.0%	0.228
Others			
Urinary tract infection (UTI)	3.1%	4.8%	0.256
Dementia	0.9%	4.8%	0.018
Ulcerative colitis	0.4%	0.0%	0.299

**Table 2:** Multivariate analysis of the 4495 combinations of 31 regulatory molecules used to predict ICU admission/Exitus in COVID-19 patients. For each molecule, the percentage of models in which the given molecule was significant (at 10%) and the average AUC contribution to ICU/Exitus prognosis calculated according to the clinical score are indicated. Also indicated are the selected molecules for the final prognostic score.

Group	Soluble Molecule	Models with Significance	Average AUC Gain	Inclusion in Final Model
Adaptive immune response	IL10	98.2%	2.0%	YES
	IFNg	69.0%	1.3%	NO
	sIL2Ra	40.0%	0.5%	NO
	sIL4R	12.9%	0.2%	NO
	IL9	0.2%	0.0%	NO
Growth factors	GCSF	100.0%	4.3%	YES
	MCSF	96.3%	2.7%	YES
	IL3	72.6%	1.1%	NO
	IL2	86.9%	1.0%	NO
	sEGFR	56.6%	0.7%	NO
	GMCSF	37.2%	0.3%	NO
	FLT3L	15.2%	0.1%	NO
	IL7	0.0%	0.0%	NO
	sVEGFR3	0.0%	0.0%	NO
Innate/inflammatory immune response	IL6	100.0%	3.5%	YES
	IL15	100.0%	3.5%	YES
	sRAGE	100.0%	3.4%	YES
	IP10	99.8%	3.2%	YES
	MCP3	98.4%	2.9%	YES
	sIL1RII	100.0%	2.4%	YES
	IL8	97.5%	2.0%	YES
	MCP1	80.5%	1.9%	NO
	TNFa	77.2%	1.8%	NO
	sTNFRII	77.0%	1.5%	NO
	MIG	63.0%	1.1%	NO
	IL1RA	70.1%	0.8%	NO
	MIP1a	29.7%	0.3%	NO
	sIL6R	4.8%	0.1%	NO
	MIP1b	0.7%	0.0%	NO
	IL27	0.7%	0.0%	NO
	EOTAXIN	0.2%	0.0%	NO

## Data Availability

The data used to support the findings of the present study are available from the corresponding author upon request.

## Supplementary Materials

The following are available online at https://www.mdpi.com/article/10.3390/biomedicines9111675/s1, Supplementary Material File SI–SVII.
