# Supplementary material for: Impact of the Innate Inflammatory Response on ICU Admission and Death in Hospitalized Patients with COVID-19
Source: Biomedicines. 2021 Nov 12;9(11):1675. doi: 10.3390/biomedicines9111675 (PMC8615389; doi:10.3390/biomedicines9111675)

Hospital Universitario Príncipe de Asturias

SaludMadrid

Comunidad de Madrid

Servicio de MEDICINA INTERNA
GUIA DE TRATAMIENTO DE LOS PACIENTES CON INFECCIÓN POR SARS-Cov-2
(Revisión 6 de Abril de 2020)

Cambios sobre la última versión:

- **Se ha retirado la parte que contemplaba el manejo de pacientes ambulatorios** ya que esta valoración se realiza por el Servicio de Urgencias donde se ha desarrollado un protocolo propio de actuación.
- **Se modifica la dosis de esteroides,** eliminando las dosis altas de los tres primeros días**.**

GUIA DE TRATAMIENTO DE LOS PACIENTES INGRESADOS CON INFECCIÓN POR SARS-Cov-2

1.- Paciente con diagnóstico clínico o microbiológico, con infiltrado radiológico, Sat de O2 < 94%, con bajos requerimientos de oxígeno (Fi O2 < 50 %, Sat O2 >92% con GN), **con** o **sin factores de riesgo**:

- **Valorar la posibilidad de su inclusión en EC de tratamiento con Remdesivir IV:** Contactar con Dr Sanz Moreno, Busca 4021. (Véase Criterios de inclusión, de exclusión y medicamentos restringidos, al final).
  - **Si es incluible, suspender Kaletra e Hidroxicloroquina y solicitar nueva PCR SARS-Cov-2 si la que tiene es de hace tres días o más.**
  - Si no es incluible o no hay disponibilidad del fármaco para el paciente, continuar como sigue.

- **LOPINAVIR/ritonavir (Kaletra®)** Comp 200/50 mg, 2 comp/12h **+ HIDROXICLOROQUINA (Dolquine®)** Comp 200 mg, dosis de carga: 2 comp/12h durante el primer día y seguido de 1 comp/12h VO] + **ACETILCISTEINA (Flumil®)** ampollas 300 mg, 600 mg/12h IV (diluido en 100ml SG5% a pasar en 15 min)

  (Actualmente no se dispone de Cloroquina).

- **Evaluación precoz de marcadores de riesgo para Síndrome de hiperactivación Inmunitaria (SHI)**
  - IL6 > 40 picogramos/ml ó
  - Al menos 2 de los siguientes:
    - Ferritina elevada (> 500)
    - D Dímero >1.5
    - PCR >150
    - Linfocitos totales < 800, o Ratio Neutrófilo/linfocito> 3.13

- **Si no hay Marcadores de riesgo para SHI, duración del tratamiento 5 días**.

- Si hay mejoría clínica y de la saturación de O2, se puede plantear el alta en condiciones de aislamiento. No es necesario repetir Rx.

- **Evaluación por su Médico de Familia.**

2.- El mismo paciente que en apartado ***1.,*** pero con mayores necesidades de O2 (Fi O2 > 50 % o Sat O2/FiO2 <315 – ver tabla al final) o mala evolución clínico-radiológica o factores de riesgo para SHI.

- **Plantear si es candidato a UCI.**

- **Añadir TOCILIZUMAB* (Roactemra®)** Vial 200 mg/10 ml, Dosis única 600 mg en 100 ml SSF a pasar en 1h.

  - **Descartar la existencia de infección bacteriana activa, antes de instaurar el tratamiento con Tocilizumab.** (incluyendo valores de procalcitonina) y **no debe tener contraindicaciones**
  - Se admite la posibilidad de tratar con Tocilizumab a pacientes no subsidiarios de tratamiento en UCI solo por edad, pero con buen estado basal y sin comorbilidades relevantes.

- Si no mejora en 24h, y el paciente no es candidato a UCI o su traslado a esta va a demorarse, o no se dispone de Tocilizumab:
  Añadir 6METIL-PREDNISOLONA: 1-2 mg/kg de peso i.v. durante 7 días, manteniendo antivíricos. A criterio clínico utilizar una dosis inicial de dexametasona de 80-160 mg i.v. en pacientes con claro empeoramiento radiológico o afectación radiológica extensa inicial.
  Mantener antivíricos mientras esté con esteroides, salvo contraindicación o efectos secundarios graves

3.- Paciente con criterios de UCI.

- En los pacientes de UCI, además de la ventilación mecánica invasiva se incluirá el tratamiento del apartado ***2.***

NOTAS:

NO UTILIZAR NEBULIZADORES. Se admite la utilización de inhaladores presurizados preferentemente con cámara intermedia.

SONDA NASOGÁSTRICA: En caso necesario se administrará LOPINAVIR/RITONAVIR SOLUCIÓN ORAL 80/20 (mg/ml) 5 ml /12h. Se utilizará una sonda **que no sea de material de poliuretano**. Utilizar sonda de PVC.

PACIENTES CANDIDATOS A UCI: Ver criterios emitidos por dicho Servicio.

ANTIBIOTERAPIA: Solo si sospecha de infección bacteriana (incluyendo valores de procalcitonina elevados). Ceftriaxona, Azitromicina o Levofloxacino

HEPARINA (HBPM) PROFILÁCTICA, en todos los pacientes. Inhixa 40 mg SC/día (ajuste según peso corporal).

ANTICOAGULACIÓN PLENA: Ver Protocolo de Tromboprofilaxis y Anticoagulación en COVID.

TOCILIZUMAB. (Roactemra®) Vial 200 mg/10 ml, Dosis única 600 mg en 100 ml SSF a pasar en 1h. Pacientes con peso < 75 Kg: Dosis 400 mg = 2 viales; > 75 Kg: Dosis 600 mg = 3 viales.

Contraindicaciones.
Sospecha de coinfección bacteriana (clínica, procalcitonina elevada, ...) y/o sepsis por otros patógenos distintos de COVID-19.
- AST/ALT >10 veces valor de referencia
- Neutrófilos < 500
- Plaquetas < 50.000
- Embarazo
- Antecedentes de diverticulitis
- Baja expectativa de supervivencia según criterios de enfermo crítico.

OTROS MODULADORES DE LA INMUNOINFLAMACIÓN: Actualmente no hay evidencia disponible para su uso. Se planteará según la llegada de nuevas evidencias

2.3. CÁLCULO DE COCIENTE SatO2/FiO2

FiO_2	Saturación periférica de oxígeno									
	85%	88%	90%	92%	94%	95%	96%	97%	98%	99%
21%	238	262	286	310	333	357	381	429	476	524
24%	208	229	250	271	292	313	333	375	417	458
26%	192	212	231	250	269	288	308	346	385	423
28%	179	196	214	232	250	268	286	321	357	393
31%	161	177	194	210	226	242	258	290	323	355
35%	143	157	171	186	200	214	229	257	286	314
40%	125	138	150	163	175	188	200	225	250	275
45%	111	122	133	144	156	167	178	200	222	244
50%	100	110	120	130	140	150	160	180	200	220
60%	83	92	100	108	117	125	133	150	167	183
70%	71	79	86	93	100	107	114	129	143	157
80%	63	69	75	81	88	94	100	113	125	138
90%	56	61	67	72	78	83	89	100	111	122
100%	50	55	60	65	70	75	80	90	100	110

Hospital Universitario Príncipe de Asturias

SaludMadrid

Comunidad de Madrid

ENSAYOS CLÍNICOS CON REMDESIVIR EN INFECCIÓN POR COVID-19

Inclusion Criteria

1) Willing and able to provide written informed consent prior to performing study procedures
2) Aged ≥ 18 years or aged ≥ 12 and < 18 years of age weighing ≥ 40 kg (where permitted/approved)
3) SARS-CoV-2 infection confirmed by PCR test ≤ 4 days before randomization *
4) Currently hospitalized (moderate only: and requiring medical care for COVID-19)
5) SpO2 ≤ 94% on room air or requiring supplemental oxygen at screening (severe) **
5) SpO2 > 94% on room air at screening (moderate)
6) Radiographic evidence of pulmonary infiltrates
7) Men and women of childbearing potential who engage in heterosexual intercourse must agree to use protocol specified method(s) of contraception

* Nasal swab, oral pharyngeal and stool are acceptable mechanisms for PCR detection

** Prefer oxygenation constant over several hours at screening, but is up to clinician determination

Exclusion Criteria

1) Participation in any other clinical trial of an experimental treatment for COVID-19
2) Concurrent treatment with other agents with actual or possible direct acting antiviral activity against SARS-CoV-2 is prohibited < 24 hours prior to study drug dosing
3) Evidence of multiorgan failure (severe)
4) Mechanically ventilated (including V-V ECMO) ≥ 5 days, or any duration of V-A ECMO (severe)
5) Requiring mechanical ventilation at screening (moderate) (CPAP is accepted)
6) ALT or AST > 5 x ULN
7) Creatinine clearance < 50 mL/min (Cockcroft-Gault for ≥ 18 yo and Schwartz for < 18 yo)
8) Positive pregnancy test
9) Breastfeeding woman
10) Known hypersensitivity to the study drug, the metabolites, or formulation excipient

Prior and Concomitant Medications

Concomitant use of the following is prohibited in participants receiving RDV and needs to be discontinued at minimum 24 hours prior to receiving first dose of study treatment:

- Traditional herbal treatments including herb sho-saiko-to (or Xiao-Shai-Hu-Tang)
- Investigational agents for COVID-19 including approved HIV protease inhibitors such as lopinavir/ritonavir, chloroquine, interferon, etc.
- Concomitant use of investigational agents such as approved HIV protease inhibitors like lopinavir/ritonavir, chloroquine, interferon, etc while receiving RDV is prohibited due to lack of evidence on additive or synergistic effects and potential for an increased risk of transaminase elevations.
- Medications will be assessed only from Day 1 prior to enrollment to Day 14 or discharge, whichever is earlier.

ALGORITMO TERAPEUTICO

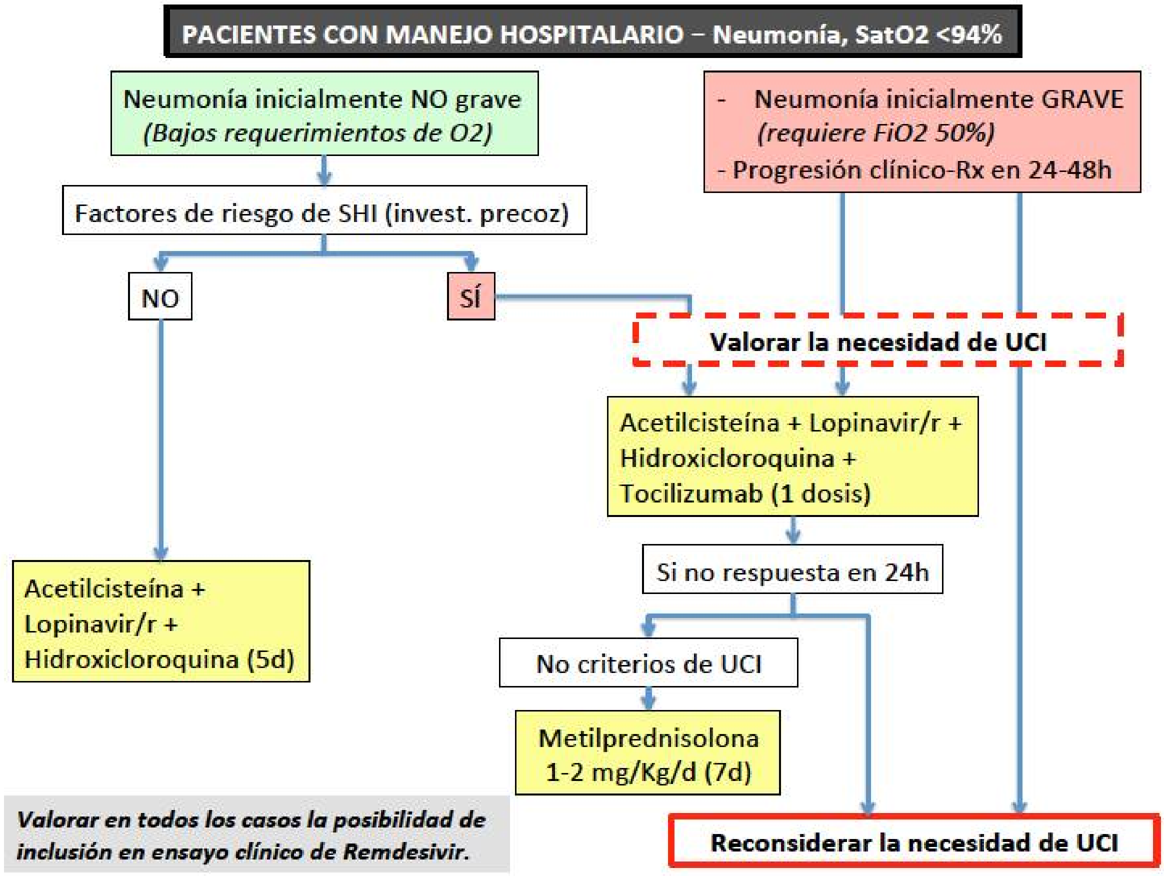

Supplement: Supplementary file 1 [file biomedicines-09-01675-s001.zip › SM I.pdf]
