# Supplementary material for: Impact of the Innate Inflammatory Response on ICU Admission and Death in Hospitalized Patients with COVID-19
Source: Biomedicines. 2021 Nov 12;9(11):1675. doi: 10.3390/biomedicines9111675 (PMC8615389; doi:10.3390/biomedicines9111675)
Supplement: Supplementary file 1 [file biomedicines-09-01675-s001.zip › SM II.pdf]

IL22= Interleukin 22

sCD40L= soluble (s) CD40 ligand (L),

IL17E/IL25= Interleukin 17E o IL-25 (Son la misma citoquina)

sCD30= soluble CD30

sgp130= soluble gp130

IFNa2= Interpheron alpha 2

IL13= Interleukin- 13

IL9= Interleukin- 9

TNFβ= Tumour necrosis factor β (También llamado LTA)

IL5= Interleukin-5

IL4= Interleukin- 4

IL17A= Interleukin- 17A (También llamado CTLA8)

sIL4R= soluble Interleukin- 4 receptor

IL17F= Interleukin- 17F (También llamada ML-1)

IL10= Interleukin-10

sIL1RI= soluble Interleukin-1 receptor type I

IFNg= Interferon gamma

TGFa= Transforming growth factor alpha (TGF-α)

sIL2Ra= soluble Interleukin-2 receptor alpha

IL3= Interleukin-3

PDGFBB= Platelet Derived Growth Factor-BB

PDGFAA= Platelet Derived Growth Factor-aa

sVEGFR1= soluble (s) vascular endothelial growth factor (VEGF) receptor 1 (También llamado sFLT-1)

FGF2= fibroblast growth factor-2

FLT3L= FMS-like tyrosine kinase 3 ligand

sVEGFR2= soluble (s) vascular endothelial growth factor (VEGF) receptor 2

sVEGFR3= soluble (s) vascular endothelial growth factor (VEGF) receptor 3

GMCSF= Granulocyte-macrophage colony-stimulating factor

VEGFA= Vascular endothelial growth factor A

IL2= Interleukin.2

EGF= Epidermal growth factor

GCSF= Granulocyte colony-stimulating factor (También llamado CSF-3)

IL7= Interleukin-7

MCSF= Macrophage colony-stimulating factor (También llamado CSF-1)

MDC= Macrophage-derived chemokine (también llamado CCL22)

IL12p70 = Heterodimeric (activated) Interleukin-12 (No hay traducción como tal p70 indica que es un heterodímero activo,

IL12p40= Homodimeric (inactivated) IL-12 (p40 indica IL-12 en forma de homodímero, inactivo)

IL18= Interleukin-18

FRACTALKINE (También llamado CX3CL1 - Chemokine (C-X3-C motif) ligand 1)

IL27= Interleukin- 27

MIP1a= macrophage inflammatory protein 1-alpha (También se llama Chemokine (C-C motif) ligand 3 (CCL3))

MIP1beta= macrophage inflammatory protein 1-beta

IL1b= Interleukin-1 beta

sIL6R= secreted Interleukin 6 receptor

TNFa= Tumour necrosis factor alpha

sRAGE= soluble (receptor for advanced glycation endproducts),

IL1RII= Interleukin 1 receptor type II

IP10= Interferon gamma-induced protein 10 (También llamado CXCL10)

IL1a= Interleukin 1 alpha

MCP1= monocyte chemoattractant protein 1 (También llamado CCL2)

IL6= Interleukin 6

IL1RA= Interleukin-1 receptor alpha

RANTES= Regulated upon Activation, Normal T Cell Expressed and Presumably Secreted (También llamado CCL-5)

IL15= Interleukin-15

sTNFRI= Soluble tumor necrosis factor receptors 1

sTNFRII= Soluble tumor necrosis factor receptors 2

MIG= monokine induced by gamma interferon (CXCL9)

IL8= Interleukin-8
