# Supplementary material for: Impact of the Innate Inflammatory Response on ICU Admission and Death in Hospitalized Patients with COVID-19
Source: Biomedicines. 2021 Nov 12;9(11):1675. doi: 10.3390/biomedicines9111675 (PMC8615389; doi:10.3390/biomedicines9111675)
Supplement: Supplementary file 1 [file biomedicines-09-01675-s001.zip › SM III.pdf]

Statistical Plan Analysis

Univariate analysis

For each cytokine, we define a dummy variable indicating whether the level of the patient is above or below the optimal threshold to predict the binomial target UCI or Exitus following the criterion based on Youden's Index [1-3]. We calculate the odds ratio of each cytokine as the odds ratio of its dummy variable, with respect the binomial target UCI or Exitus.

Multivariate analysis

As highlighted in Levine and Renelt [4] and Sala-i-Martin [5], as soon as one starts combining multivariate regressions combining the various variables (cytokines, in our case), one finds out that a certain variable x_i is significant when the model includes x_j and x_k, but it becomes non-significant when x_z is included. The problem, known as "Nothing is robust", is exacerbated when the sample size is just one order of magnitude above the total number of explanatory variables. Since we don't know a priori which are the "true" variables that should be included in the model, we are left with the question: what are the cytokines that are really correlated with fatal outcome of COVID-19 patients?

To answer this question, we follow a strategy based on the "extreme bounds" test [6,7] and the distribution test [5]. In short, given *J* potential explanatory variables, to test the robustness of the *J*-th variable, the "extreme bounds" test estimates all the potential models using the *J*-th variable and *k* other variables - $\binom{J-1}{k}$ models-. Each model provides an estimation of the interval of confidence of the parameter, $\beta_{J,m} \mp \tau_{1-\frac{\alpha}{2}} \sigma_{J,m}$, where $\beta_{J,m}$ is the estimated value of the parameter corresponding to the *J*-th variable in the *m* model, $\sigma_{J,m}$ its standard deviation, and $\tau_{1-\frac{\alpha}{2}}$ the value of the t-student distribution with *N*-

k degrees of freedom, for a signification α. If the interval of confidence built with the lowest lower bound and the highest upper bound is strictly positive or strictly negative, the variable is said to be robust. That is, the *J*-th variable is said to be robust if it is statistically significant in all the models, and the parameter has always the same sign. The test is so strict that typically leads to non-robustness. Hence, a modification was suggested by Sala-i-Martin [5] to account for the distribution of the $\binom{J-1}{k}$ estimates of the parameter associated with the *J*-th variable. Instead of having a binary answer to the robustness problem, a level of confidence in the robustness of the parameter is given, based on the distribution of the estimates.

In our case, the target is a binomial variable that equals 1 if the patient was an "Exitus" or treated in an ICU. We fix an explanatory variable that consists of a risk score based on historical records and oxygen saturation. The score was estimated using the EM-7 model by Alvarez-Mon et al. [8], which was trained using a population of 3.247 COVID-19 patients in Spain, and achieved and AUC of 78,41%. The risk score alone has an AUC of 81,13% on the selected 287 patients of these analysis. On top of the risk score, we let each model to introduce three cytokines at the same time. Hence, $\binom{N}{3}$ models will be built, where N is the number of selected cytokines.

We introduce two modifications to the distribution test approach. First, we use Bayesian logistic models, where no fixed distribution is assumed on the parameters (GLM estimation assumes normal distribution). Low sample size can lead to less robust estimations of parameters and standard errors, thus compromising the GLM significance test, which relies on asymptotic properties of the estimators [9] We perform the Bayesian estimation using the *brms package* available in R [10] and using no prior to avoid introducing any bias.

Second, apart from the signification, we estimate the marginal contribution that each variable has in the output of the models. We estimate the relative importance of each variable included in the model using SHAP (SHapley Additive ExPlanation) values [29,30]. Given an observation $x = (x_1, \dots, x_J)$, the SHAP value of feature j on instance x corresponds to the modification that the concrete value of feature j on x makes to the output of the model, with respect to other instances that share some features with x but not j. For a parametric model $F(x) = g(\sum_j \alpha_j x_j)$, where g is a function of the weighted features of x, the SHAP value corresponds to: $\varphi_j(x) = \alpha_j(x_j - E(X_j))$ where X is the set of observations and $E(X_j)$ is the average value of the j feature on X. Then, noting as n the total number of observations, we can estimate the relative importance of feature j in the model as:

$$RI(j) = \frac{\sum_{i=1}^{n} |\varphi_j(x_i)|}{\sum_{k=1}^{J} \sum_{i=1}^{n} |\varphi_k(x_i)|}$$

Formula 1. Relative importance

The average contribution of a cytokine is defined as the AUC gain attributable to the cytokine, with respect a model that consists only of the risk score based on medical record and oxygen saturation. Noting by x_{c1}, x_{c2}, x_{c3} the three cytokines included in a model m, and by x_r the risk score, the AUC gain of x_{c1} is:

$$AUCgain(x_{c1}) = \frac{RI(x_{c1})}{RI(x_{c1}) + RI(x_{c2}) + RI(x_{c3})} (AUC(m) - AUC(x_r))$$

Formula 2. Individual AUC gain.

References:

1. Aoki K, Misumi J, Kimura T, Zhao W, Xie T Evaluation of Cutoff Levels for Screening of Gastric Cancer Using Serum Pepsinogens and Distributions of Levels of Serum Pepsinogens I, II and of PG I/PG II Ratios in a Gastric Cancer Case-Control Study. *J. of Epidemiol*., **1997**, 7, 143–151.
2. Greiner M, Pfeiffer D, Smith RD. Principals and Practical Application of the Receiver Operating Characteristic Analysis for Diagnostic Tests." *Prev. Vet. Med.* **2000**, 45, 23–41
3. Youden WJ. Index for Rating Diagnostic Tests. *Cancer*, **1950**, 3, 32–35.
4. Levine, R., & Renelt, D. A Sensitivity Analysis of Cross-Country Growth Regressions. *Am. Econ. Rev*, **1992**, 82(4), 942-963.
5. Sala-i-Martin, X. I Just Ran Two Million Regressions. *Am. Econ. Rev*, **1997**. 87, 178-183.
6. Leamer, E. Let's Take the Con Out of Econometrics, *Am. Econ. Rev*., **1983**, 73, 31-43
7. Leamer, E. Sensitivity Analyses Would Help. *Am. Econ. Rev*, **1985**, 75, 308-313.
8. Álvarez-Mon, M.; Ortega, M.A.; Gasulla, Ó.; Fortuny-Profitós, J.; Mazaira-Font, F.A.; Saurina, P.; Monserrat, J.; Plana, M.N.; Troncoso, D.; Moreno, J.S.; et al. A predictive model and risk factors for case fatality of covid-19. *J. Pers. Med.* **2021**, 11, 1–18, doi:10.3390/jpm11010036.
9. Western, B., and Jackman, S. Bayesian Inference for Comparative Research. *Am. Pol. Sci. Rev.* **1994**, 88, 412-423.
10. Bürkner, Paul-Christian. brms: An R Package for Bayesian Multilevel Models Using Stan. *J. Stat. Softw,* **2017**, 80, 1-28.
