# Supplementary material for: Impact of the Innate Inflammatory Response on ICU Admission and Death in Hospitalized Patients with COVID-19
Source: Biomedicines. 2021 Nov 12;9(11):1675. doi: 10.3390/biomedicines9111675 (PMC8615389; doi:10.3390/biomedicines9111675)
Supplement: Supplementary file 1 [file biomedicines-09-01675-s001.zip › SM IV.pdf]

Table S1. Description of the innate inflammatory and adaptive IR and growth factors cytokine in blood from COVID-19 patients. The table presents for each molecule analyzed, the percentage of population that showed detectable serum levels (considering as 0 those who did not show it) and the mean and SD serum level (pg/ml) for both COVID-19 patients, and healty controls and the results of the t-test between both average levels. For patients with more than one sample, only the first sample is considered.

**Group**	**Soluble molecules**	**Healthy patients**		**COVID patients**		**p-value of the t-test**
		**% that showed**	**Mean**	**% that showed**	**Mean**	**%**
Adaptive immune response	IL22	42,9%	43,3	47,0 %	129,6	83,8 %
	sCD40L	100,0%	5514,4	99,7 %	6871,5	66,9 %
	IL17EIL25	100,0%	717,2	99,3 %	977,9	62,0 %
	sCD30	71,4%	66,0	61,7 %	98,8	41,1 %
	sgp130	100,0%	146680,2	99,7 %	168769,2	18,3 %
	IFNa2	100,0%	108,7	99,0 %	175,0	10,1 %
	IL13	100,0%	79,0	99,3 %	149,4	2,8 %
	IL9	100,0%	21,7	99,3 %	31,5	1,0 %
	TNFb	92,9%	11,4	99,3 %	39,8	0,9 %
	IL5	100,0%	9,9	100,0 %	21,7	0,5 %
	IL4	92,9%	2,5	97,9 %	8,7	0,2 %
	IL17A	92,9%	8,7	97,6 %	26,2	0,1 %
	sIL4R	100,0%	868,9	100,0 %	1606,8	0,0 %
	IL17F	7,1%	3,3	19,9 %	47,1	0,0 %
	IL10	64,3%	7,2	89,9 %	57,6	0,0 %
	sIL1RI	0,0%	0,0	14,3 %	11,4	0,0 %
	IFNg	92,9%	7,6	97,2 %	52,3	0,0 %
	TGFa	85,7%	5,8	99,3 %	27,0	0,0 %
	sIL2Ra	100,0%	298,5	99,3 %	1346,0	0,0 %
Growth factors	IL3	42,9%	0,8	20,2 %	1,2	7,4 %
	PDGFABBB	100,0%	50603,2	100,0 %	78446,5	1,7 %
	sEGFR	100,0%	33318,6	99,3 %	43476,5	1,3 %
	PDGFAA	100,0%	6020,6	100,0 %	11711,2	0,2 %
	sVEGFR1	21,4%	91,7	39,0 %	438,7	0,1 %
	FGF2	92,9%	107,7	99,3 %	182,6	0,0 %
	FLT3L	100,0%	21,2	99,7 %	43,4	0,0 %
	sVEGFR2	100,0%	9512,8	99,3 %	14522,6	0,0 %
	sVEGFR3	100,0%	858,2	94,8 %	1914,3	0,0 %
	GMCSF	14,3%	0,6	18,5 %	9,2	0,0 %
	VEGFA	100,0%	280,0	100,0 %	993,8	0,0 %
	IL2	64,3%	1,2	86,4 %	5,9	0,0 %

	EGF	92,9%	67,8	99,3 %	294,0	0,0 %
	GCSF	92,9%	28,2	85,0 %	329,5	0,0 %
	IL7	100,0%	6,7	98,6 %	32,2	0,0 %
	MCSF	64,3%	107,7	94,4 %	766,9	0,0 %
Innate and inflammatory immune response	MDC	100,0%	1183,5	100,0 %	1112,5	38,6 %
	IL12p70	100,0%	7,1	78,0 %	24,2	37,8 %
	IL18	100,0%	66,9	98,6 %	87,9	33,4 %
	FRACTALKINE	100,0%	264,8	100,0 %	345,7	20,2 %
	IL27	100,0%	5088,8	100,0 %	7188,4	6,1 %
	IL12p40	100,0%	66,2	99,3 %	133,8	0,1 %
	MIP1a	100,0%	35,2	98,3 %	61,3	0,1 %
	MCP3	100,0%	48,7	100,0 %	105,4	0,1 %
	IL1b	92,9%	16,5	99,3 %	46,9	0,0 %
	EOTAXIN	100,0%	161,7	100,0 %	329,4	0,0 %
	MIP1b	100,0%	43,8	99,7 %	79,6	0,0 %
	GROa	92,9%	26,1	99,0 %	100,3	0,0 %
	sIL6R	100,0%	16112,1	99,3 %	30030,6	0,0 %
	TNFa	100,0%	50,8	100,0 %	125,8	0,0 %
	sRAGE	78,6%	36,2	88,5 %	137,1	0,0 %
	sIL1RII	100,0%	4983,3	99,7 %	11880,4	0,0 %
	IP10	100,0%	404,8	99,7 %	6469,8	0,0 %
	IL1a	85,7%	17,4	93,7 %	75,0	0,0 %
	MCP1	100,0%	679,2	100,0 %	2497,0	0,0 %
	IL6	100,0%	4,0	99,7 %	662,7	0,0 %
	IL1RA	100,0%	7,4	97,9 %	49,7	0,0 %
	RANTES	100,0%	3522,4	99,7 %	8015,8	0,0 %
	IL15	100,0%	12,9	99,7 %	47,7	0,0 %
	sTNFRI	100,0%	520,9	98,6 %	1937,8	0,0 %
	sTNFRII	100,0%	3641,1	99,7 %	12665,3	0,0 %
	MIG	100,0%	2352,6	100,0 %	11596,5	0,0 %
	IL8	100,0%	8,2	99,7 %	72,6	0,0 %
