# Supplementary material for: Impact of the Innate Inflammatory Response on ICU Admission and Death in Hospitalized Patients with COVID-19
Source: Biomedicines. 2021 Nov 12;9(11):1675. doi: 10.3390/biomedicines9111675 (PMC8615389; doi:10.3390/biomedicines9111675)
Supplement: Supplementary file 1 [file biomedicines-09-01675-s001.zip › SM V.pdf]

Table S2. Odds ratio for ICU or death of the innate inflammatory and adaptive IR and growth factors cytokine measured in blood por severe COVIV19 patients. For each of the 52 cytokine, it is presented the odds ratio of its dummy variable, with respect the binomial target UCI or Exitus and the p-value of significance of the odds ratio being equal to 1. OR = odds ratio

Group	Smolecules	OR	p-value
Adaptive immune response	IL10	9,9	0,0 %
	sIL2Ra	3,3	0,0 %
	IL17A	3,0	0,2 %
	IFNg	2,6	0,1 %
	IL4	2,5	2,8 %
	IL13	2,4	4,5 %
	sIL4R	2,3	0,6 %
	TNFb	2,1	1,9 %
	sIL1RI	1,9	10,9 %
	IL9	1,8	3,5 %
	IL17F	1,6	16,0 %
	IL5	1,6	23,8 %
	TGFa	1,5	19,0 %
Growth factors	GCSF	8,9	0,0 %
	MCSF	5,3	0,0 %
	FGF2	4,5	0,0 %
	GMCSF	3,5	0,2 %
	IL3	2,6	0,6 %
	IL2	2,5	0,2 %
	sEGFR	2,3	5,3 %
	IL7	2,2	1,8 %
	FLT3L	1,8	3,8 %
	sVEGFR3	1,7	6,4 %
	sVEGFR1	1,2	51,4 %
	sVEGFR2	1,2	65,5 %
	VEGFA	1,2	76,1 %
	EGF	1,1	93,0 %
	PDGFABBB	0,5	62,5 %
	PDGFAA	0,4	17,9 %
Innate and inflammatory immune response	IL6	8,0	0,0 %
	IL15	6,7	0,0 %
	MCP3	5,9	0,0 %
	sIL1RII	5,4	0,0 %
	IP10	5,0	0,0 %

TNFa	4,7	0,0 %
sTNFRII	4,3	0,0 %
IL8	4,2	0,0 %
sRAGE	4,2	0,0 %
MIG	4,2	0,0 %
sTNFRI	3,8	0,0 %
MCP1	3,4	0,0 %
IL1b	2,9	0,1 %
IL1RA	2,8	0,0 %
IL27	2,5	0,4 %
EOTAXIN	2,4	6,3 %
sIL6R	2,3	0,5 %
MIP1a	2,2	0,7 %
IL1a	2,0	1,8 %
MIP1b	1,8	7,7 %
GROa	1,6	10,1 %
IL12p40	1,4	22,5 %
RANTES	1,2	86,8 %
